# In silico metabolic modelling links microbiome-derived metabolites to risk factors of Alzheimer’s disease

**DOI:** 10.1080/29933935.2024.2443171

**Published:** 2024-12-23

**Authors:** Tim Hensen, Shahzad Ahmad, Gabi Kastenmüller, Robert Kraaij, Mohsen Ghanbari, Arfan Ikram, Rima Kaddurah-Daouk, Ines Thiele

**Affiliations:** a Digital Metabolic Twin Centre, University of Galway, Galway, Ireland; b School of Medicine, University of Galway, Galway, Ireland; c Department of Epidemiology, Erasmus Medical Center, Rotterdam, The Netherlands; d Institute of Computational Biology, Helmholtz Zentrum München – German Research Center for Environmental Health, Neuherberg, Germany; e Department of Internal Medicine, Erasmus MC University Medical Center, Rotterdam, The Netherlands; f Department of Psychiatry and Behavioral Sciences, Duke University, Durham, NC, USA; g Duke Institute of Brain Sciences, Duke University, Durham, NC, USA; h Department of Medicine, Duke University, Durham, NC, USA; i School of Microbiology, University of Galway, Galway, Ireland; j Ryan Institute, University of Galway, Galway, Ireland; k APC Microbiome Ireland, University College Cork, Cork, Ireland

**Keywords:** Gut microbiome, metabolic modeling, Alzheimer’s disease, *APOE*, bile acid metabolism

## Abstract

The gut microbiome has become increasingly recognized for its role in the pathogenesis of Alzheimer’s disease (AD) and is thought to influence AD pathogenesis via metabolic crosstalk with the host. However, mechanistic pathways connecting the gut microbiome to AD pathogenesis remain unknown. To explore potential mechanistic pathways in AD pathogenesis, we created host–microbiome whole-body metabolic models personalized with 16S rRNA microbiome data and predicted emergent metabolic contributions of gut microbiomes. We analyzed 63 metabolites in blood with previously known links with AD. These *in silico* predictions were then associated with major risk factors for AD in a cohort of 1,065 aging non-AD individuals and subsequently used to inform targeted analyses on serum metabolomics. Our analysis identified increased host-microbial production of L-arginine in older individuals. Lower production of deoxycholate correlated with the neuroprotective *APOE* E2 allele and it decreased with higher global cognition. Serum metabolomics from the same individuals of cholesterol products and bile acid metabolism corroborated the modeling predictions, suggesting a potential link between the *APOE* genotype and cognitive health. In conclusion, this study associated metabolic gut microbiome influences on human metabolism with risk factors for AD and identified cholesterol and bile acid metabolism to potentially link with AD pathogenesis.

## Introduction

Alzheimer’s disease (AD) is the most prevalent form of dementia, characterized by progressive neuronal loss^1^ accumulation of amyloid-β and hyperphosphorylated tau proteins^2–4^ and widespread metabolic perturbations.^5–7^ The prevalence of AD is rising worldwide due to aging societies.^8,9^ Hence, there is a pressing need for early diagnosis and intervention strategies to delay and hold the progression of AD.^10^

The onset of AD is influenced by a wide spectrum of phenotypic and genetic risk factors,^11^ including age,^12,13^ sex,^14^ midlife obesity,^15,16^ education level,^17–19^ cognitive reserve,^20^ the alipoprotein E gene^21,22^ (*APOE*), and subjective or mild cognitive impairments.^23,24^ The gut microbiome, a large collection of microbes inhabiting the human gut, has also garnered increasing interest for its role in AD.^25–29^ The gut microbiome is known to influence the brain via a set of bidirectional biochemical pathways that form together the gut–brain axis.^30^ Communication throughout the gut-brain axis is facilitated by both host- and microbe-derived metabolites.^31^ Several groups of microbe-derived metabolites have been linked to AD, including neurotransmitters,^32–34^ short-chain fatty acids,^35^ branched-chain amino acids,^36,37^ polyamines,^38^ and bile acids.^26,39^ Hence, compositional changes in the gut microbiome may serve as early preclinical markers of^40^ and could provide a potential treatment option using noninvasive dietary changes, probiotics, and antibiotics.^41,42^

Bile acids have been of particular interest in recent efforts linking the gut-brain axis with AD pathogenesis.^43^ They have been traditionally recognized for their role in lipid absorption,^44^ but they also have antimicrobial properties,^45,46^ and play roles as signaling molecules in glucose homeostasis^47^ and in the immune system.^48^ Primary bile acids are synthesized by human metabolism in the liver and the brain as downstream products of cholesterol clearance and degradation.^49^ Cholate and chenodeoxycholate, the main primary bile acids, are detoxified via glucuronidation or taurination, after which they are transported to the colon via the biliary tract.^49^ There, specialized bacteria, including *Eggerthella lenta* ^50^ and the Clostridiaceae family,^51^ can convert these primary conjugated bile acids to secondary bile acids via various deconjugation, dehydroxylation, and epimerisation conversions.^52^ Decreased cholesterol elimination^53,54^ and increased microbial production of secondary bile acids are known to be related to AD progression and cognitive impairments.^26,55^ Based on these findings, the microbiome-bile acid-brain axis has been proposed as a potential determinant in AD pathogenesis.^27^

Despite these advances in understanding the role of the gut microbiome in AD pathogenesis, the underlying mechanisms linking host–gut interactions to AD remain unknown. Inferring such mechanistic insights can, however, be difficult using correlation-based statistical analyses alone. Thus, a non-statistical modeling approach is needed to generate mechanistic hypotheses on the gut-brain axis that can be statistically tested. One modeling approach with a proven track record to generate mechanistic hypotheses on the gut-brain axis^29,39,56,57^ is the constrained-based genome-scale reconstruction and analysis (COBRA) approach.^58^ COBRA is a computational framework for the generation and deterministic interrogation of genome-scale metabolic reconstructions. These reconstructions represent knowledge bases of metabolism of their target organism, as they are assembled based on an organism’s genome annotation, biochemical, genetic, and physiological data.^59^ Through the integration of multi-omics data, including metabolomic,^60^ microbiome,^61^ and dietary information,^62,63^ COBRA reconstructions can be converted into condition-specific genome-scale metabolic models (GEMs). Such condition-specific GEMs can be subsequently investigated by calculating fluxes through metabolic reactions in the GEMs using constrained-based optimization techniques, such as flux balance analysis (FBA).^64^

Recent innovations in the field of COBRA modeling—^61,65,66^ have enabled large-scale modeling efforts of host-microbiome co-metabolism. Extensive resources of high-quality metabolic reconstructions of gut microbes are now available, including AGORA^67^ (818 reconstructions), its successors AGORA2^68^ (7,302 reconstructions), and APOLLO^69^ (247,092 reconstructions). These resources contain strain resolved reconstructions, enabling strain-resolution modeling studies. However, higher taxonomic levels can be investigated by combining strain-level metabolic reconstructions into pan-reconstructions, which contain the metabolic contents of the combined strain-level reconstructions. Together with the introduction of computational modeling toolboxes, such as the COBRA toolbox,^58^ Microbiome Modeling toolbox,^61,70^ and MICOM,^71^ AGORA and its successors have enabled a semi-automatic workflow for the generation and interrogation of personalized microbiome community models.^72,73^ Microbiome community modeling has been applied to accurately predict metabolite concentrations in the feces^74^ and has successfully contributed to new insights on the role of the gut microbiome in various complex diseases, including Parkinson’s disease,^56,57^ inflammatory bowel disease,^51,75^ type 2 diabetes,^76^ colorectal cancer,^77^ and recently AD.^29^ The introduction of sex-specific and organ-resolved whole-body metabolic models (WBMs) further extended this microbiome modeling paradigm by enabling investigations on the metabolic host–microbiome co-metabolism.^29,65,78,79^

In this study, we aimed to link host-microbiome co-metabolism to major risk factors for AD, including age, *APOE* genotype, global cognition, and sex. To explore these microbial links, microbiome-WBMs were generated by contextualizing sex-specific WBMs with microbiome community models from gut 16S rRNA microbiome samples, which were obtained from a cross-sectional cohort of healthy aging participants from the Rotterdam study.^80^ Metabolic fluxes were then predicted for 63 selected metabolites in the blood compartment of 1,065 microbiome-WBMs. We identified microbiome-driven metabolic links between predicted L-arginine blood levels and age, and we could associate predicted microbe-derived bile acids to age, *APOE* genotype, and global cognition. Sex-specific metabolic links with global cognition were also predicted for short-chain fatty acids, creatine, and L-arginine. These results were then contextualized by identifying the main microbial drivers of the flux predictions, finding *Parabacteriodes distansonis* to be correlated with both L-arginine and aging. *Eggerthella lenta* was further correlated with global cognition and both deoxycholate and lithocholate. Finally, we also confirmed the predicted fluxes for deoxycholate with serum metabolomic data of the same individuals. Taken together, this study describes metabolic phenotypes of the host-gut system in healthy aging individuals and links them to major risk factors of AD.

## Results

In this study, we assessed host-microbiome co-metabolism in a cohort of 1,065 participants from the Rotterdam study, a prospective cohort study that follows aging individuals to investigate determinants and consequences of aging.^80^ The cohort consisted of healthy, middle-aged to elderly participants between 51 and 92 years old (mean = 61.7 years, standard deviation; SD = 5.5, Table 1). A total of 608 women and 457 men were included. Importantly, almost all the participants were cognitively healthy (Table 1) according to mini-mental state examinations (MMSE).^81^ Only 12 individuals qualified for mild cognitive impairment, defined by an MMSE score between 21 and 25^81^. For each of the 1,065 individuals, 16S rRNA sequencing data were available^82^ and blood serum metabolomic data were obtained (Methods). Note that the stool and blood samples may not have been taken at the same time.

**Table 1.** Participant characteristics*.

Variable	N	Female	Male	p-value†
Samples	1,065	608	457	
Age	1,064	61.66 (5.38)	61.64 (5.48)	9.59e-01
BMI	1,065	27.24 (4.85)	27.62 (4.07)	1.69e-01
Education** (N)	1,062	39-261-156-150	23-87-164-182	1.17e-16
Mini mental state examination score***	1,065	28.56 (1.42)	28.51 (1.36)	5.97e-01
Letter-digit substitution score	1,065	33.2 (6.43)	31.17 (5.52)	8.2e-08
Stroop colour and word test score	1,065	43.58 (12.27)	44.32 (11.24)	3.11e-01
Word fluency score test	1,065	24.97 (5.6)	24.39 (5.62)	9.21e-02
Delayed word fluency test score	1,065	9.33 (2.65)	8.21 (2.66)	1.14e-11
Left hand Purdue pegboard score	1,065	38.39 (4.51)	35.41 (4.94)	1.33e-23
Global cognition score	1,065	0.3 (1.5)	−0.35 (1.44)	1.87e-12
APOE E2/E2 (N)	5	5	0	
APOE E2/E3 (N)	121	74	47	
APOE E2/E4 (N)	25	15	10	
APOE E3/E3 (N)	644	350	294	
APOE E3/E4 (N)	251	157	94	
APOE E4/E4 (N)	19	7	12	

*Values reported as the mean (standard deviation) unless otherwise indicated.

**The following education levels are shown from left to right: primary education, lower/intermediate general education or lower vocational education, intermediate vocational education or higher general education, higher vocational education or university education.

***The MMSE scores were not included in the global cognition score.

†P-values were calculated based on two-sample t-tests and with the Fisher exact tests of independence for education.

### Mapping of microbial taxonomies

To characterize the metabolic properties of host-microbiome co-metabolism from the 1,065 participants in the Rotterdam cohort, we created personalized microbiome-WBMs using 16S rRNA sequencing data obtained from each individual’s stool sample. In a first step, we identified all microbial species in the overall data set and removed microbial taxa of lower taxonomic resolutions. This step was necessary as lower resolution pan-reconstructions would include metabolic capabilities of species absent in the microbiomes. Filtering on the species-level, however, led to a substantial decrease in taxonomic diversity across the microbiomes (from 359 taxa to 151 taxa, Table 2, Table S1) as well as reduction in the total read count per sample to an average of 49.5% (SD = 9.7%, Table 2table:mappingStats). Subsequently, we mapped the identified species onto the strain-resolved metabolic reconstruction resource, AGORA2,^68^ and created pan-species reconstructions for each species, which contained the combined metabolic capabilities of all corresponding strains. In AGORA2, overall, 116 (77%) of the identified species were covered (Table 2, Table S1), mapping 64.0% (SD = 11.6%) of the species reads. The overall mapped reads (named AGORA2-based mapped reads) contained on average 31.9% (SD = 9.3%) of the raw, unfiltered reads.

**Table 2.** The number of identified taxa, species, and mapped species are reported.

Feature	Statistic
Total number of named taxa	359
Total number of named species	151
Total number of species mapped by AGORA2	116
Mean richness of named taxa	92.26 (SD = 22.61)
Mean richness of species-level taxa	48.50 (SD = 11.07)
Mean richness of AGORA2 mapped species	27.10 (SD = 6.59)
Mean read coverage of named species	49.53% (SD = 9.66%)
Mean read coverage of AGORA2 mapped species	31.88% (SD = 9.34%)
Mean AGORA2 mapping coverage of species-level reads	64.02% (SD = 11.63%)

The percentage of raw reads before and after mapping was calculated for each individual sample based on the provided relative read abundances. Taxa with relative abundances below 0.0001% were excluded in both calculations. The taxonomic richness was calculated by counting the number of taxa with relative abundances above 0.0001% for each individual. The AGORA2 mapping coverage of species-level reads represents the percentage of reads covered by the mapped taxa compared to all taxa identified at the species resolution. Please refer to the methods section for more details on the AGORA2 mapping procedure.

The 116 pan-species reconstructions covered seven phyla, of which *Firmicutes* and *Bacteroidota* were the most abundant phyla with mean relative abundances of 76.11% and 18.23%, respectively (Table S2). *Firmicutes* and *Bacteroidota* were also the most abundant phyla before AGORA2 mapping, with respective pre-mapped average relative abundances of 79.62% and 13.32% (Table S2). At the species level, *Faecalibacterium prausnitzii* (16.33%), *Ruminococcus bromii* (8.31%), *and Butyrivibrio crossotus* (8.22%) had the highest relative abundances (Table S3). The pan-species reconstructions were then combined to create 1,065 individual, sample-specific metabolic microbial community models, which were subsequently joined with the WBMs in a sex-specific manner (Methods, Table 3), i.e., female hosts were combined with microbiomes from female participants, while male hosts were combined with microbiomes from male participants. Further parameterization was done by specifying a dietary input corresponding to an average European diet.^62^ Note that no further host genetic, dietary, phenomenological, or exposomal information was available. Hence, the WBMs could not account for inter-individual variations in these factors. Taken together, the 16S rRNA data were used to generate individual sex-specific, microbiome-WBMs that only differed in their microbial, metabolic part, thereby allowing for the *in silico* investigation of their influence on host-microbiome metabolism and emerging host phenotypes.

**Table 3.** Summary statistics for the contents of the 1,065 microbiome personalised WBMs, microbial reaction and subsystem contents.

Statistic	Female	Male
Host reactions (N)	85,403	82,913
Host metabolites (N)	60,341	57,897
Host constraints (N)	109,148	105,335
Host organs (N)	32	30
Host subsystems (N)	104	104
Microbial reactions: Mean (SD)	49,416.14 (11,996.79)	50,935.58 (12,827.86)
Microbial subsystems: Mean (SD)	123.47 (3.15)	123.55 (2.76)
Microbiome-WBM reactions: Mean (SD)	132,871.95 (11,999.27)	131,909.58 (12,827.86)
Microbiome-WBM metabolites: Mean (SD)	101,763.59 (10,452.82)	100,662.40 (11,212.86)
Microbiome-WBM constraints: Mean (SD)	177,436.15 (18,001.87)	175,955.72 (19,293.15)

### Metabolic flux predictions

After generating the 1,065 microbiome-WBMs, microbial influences on host metabolic phenotypes were investigated by predicting the maximal possible metabolite accumulations in the blood. Therefore, we selected 67 metabolites that were present in the WBM blood compartments and had established links to cognitive decline,^73^ as well as known involvement with gut microbial metabolism (Methods). This list contained secondary bile acids, neurotransmitters, short-chain fatty acids, metabolites in methionine metabolism, and metabolites related to energy homeostasis (Table S4). FBA was performed to predict metabolite accumulation fluxes in the blood (Methods, Table S5, Table S6) for each selected metabolite in the microbiome-WBMs and in the germ-free WBMs, i.e, WBMs without integrated microbial community models. Four metabolites were excluded from further analyses due to the low number of samples for which accurate gut microbiome influences could be predicted. Two of the four metabolites were removed because no non-zero flux values could be predicted in over 90% of samples (i.e., 958 samples), while the other two metabolites were removed due to having identical flux values in over 90% of the samples (Table S4). Additionally, we found 31 stoichiometrically dependent metabolites, belonging to the same (linear) pathways. Hence, they were organized into nine distinct metabolic groups (Table S7). For the remainder of the analysis, we considered the metabolite flux predictions for the 32 metabolites and nine metabolite groups. Note that the host part of the microbiome-WBMs was identical within the sexes. Thus, any variation in the predicted metabolic fluxes was driven by variations in the gut microbial relative abundances, their associated interactions with host metabolism, and their capabilities in producing either the metabolite and/or its precursors.

### Increased microbial capacity of L-arginine production was observed in advanced age

**Figure 1.**
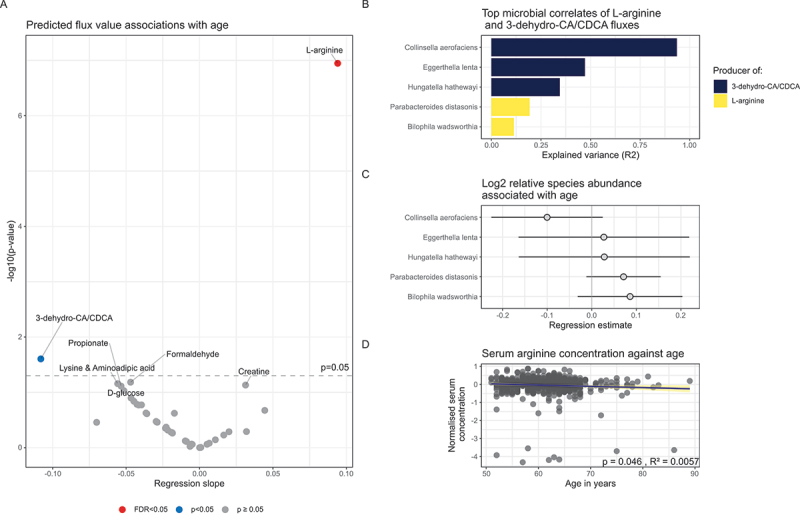
Integrated analysis of predicted L-arginine and 3-dehydro-CA/CDCA blood fluxes.

As age is a major risk factor for AD dementia and cognitive decline in general,^12,13^ we investigated whether any of the predicted metabolite fluxes could be associated with age. Therefore, we performed linear regression on age against the blood fluxes with a control variable for sex. A strong increase with age was found for the L-arginine blood. (*p* = 1.13⋅10^−7^, false discovery rate adjusted p-value (FDR) = 4.64⋅10^−6^, Figure 1(A), Table S8). As the microbiome-WBMs are mechanism-based, we could identify all those microbes that can exchange L-arginine with the large intestinal lumen of the microbiome-WBMs (Table S9), resulting in 68 candidate L-arginine producing species. The relative abundances of these 68 species were then regressed against L-arginine blood fluxes to identify which species were major drivers of the predicted L-arginine fluxes, i.e., obtained the largest R-squared ($R^{2}$) value (Table S10). The relative abundance of *Parabacteroides distasonis* ($R^{2}$= 0.19) and *Bilophila wadsworthia* ($R^{2}$ = 0.11) showed the highest correlation with the predicted L-arginine blood fluxes (Figure 1(B)). However, when performing linear regressions on the relative abundances of these 68 candidate species against age, we only found a weak positive association with age for *P. distasonis* (*p* = 0.09), and no association for the other highly correlating species (Figure 1(C), Table S11). These results suggest that the predicted increased L-arginine production capacity may be an emergent phenomenon arising from alterations in abundances of multiple species and the production of L-arginine precursors. To test if the increased gut microbial capacity to produce L-arginine would translate to increased serum metabolomic arginine concentrations, we performed a linear regression on the serum L-arginine concentrations from the matched metabolomic measurements against age, with a control variable for sex (Methods, Table S12). However, no significant association of serum L-arginine concentrations with age could be found (Figure 1(D), Table S13), suggesting a non-significant influence of age-related gut microbiome perturbations towards serum L-arginine concentrations. While this result does not further support our predictions of microbial-associated changes in blood L-arginine, it is also not rejected as other external factors, such as diet and lifestyle, could influence the measured serum L-arginine concentrations.

A) Volcano plot of regression outcomes for all 41 distributions. The regression estimates are shown in the x-axis. The y-axis displaying −log10 transformed regression p-values. The estimates indicate the coefficient of relative change in flux with increasing age. For example, positive estimates coupled with a significant p-value indicate that the microbial flux capacity increased with age. The red dot for L-arginine represents an FDR-corrected p-value below 0.05. The blue coloured dot for the 3-dehydro-CA/CDCAs indicates a nominal p-value below 0.05 while missing significance after FDR correction. Furthermore, all metabolites with regression p-values below 0.1 are labelled. B) Bar plot showing all species that explained a minimum of 10% of variance in the predicted L-arginine blood fluxes and the 3-dehydro-CA/CDCA blood fluxes and contained metabolic reactions for the production of these compounds. The dark blue coloured bars indicate species that have the metabolic capacity to produce the 3-dehydro-CA/CDCAs, while the yellow bars indicate species that can produce L-arginine. C) Forest plot of regression outcomes of species-level relative abundances against age. Lines for the 95% confidence intervals are displayed, with the middle dots indicating the regression estimate. D) Scatter plot of normalised serum arginine concentrations in the metabolomic data on the y-axis against the age in years on the x-axis. The blue regression line indicates the fitted regression line for the arginine concentrations against age. The 95% confidence interval is shown by the yellow shaded area around the regression line.

### Microbial capacity to produce 3-dehydro cholate/chenodeoxycholate decreased with age and was driven by *Collinsella aerofaciens*

In addition to L-arginine, the predicted blood metabolic fluxes for the bile acids, 3-dehydroxycholate, and 3-dehydroxy-chenodeoxycholate (metabolite group: 3-dehydro-CA/CDCA) were slightly decreased with age (*p* = 0.02, FDR = 0.51, Table S8). When tracing back the most influential species for the 3-dehydro-CA/CDCA blood fluxes, a high $R^{2}$ value was found with *C. aerofaciens* ($R^{2}$ = 0.94, Figure 1(B), Table S10) and *Eggerthella lenta* ($R^{2}$ = 0.47), suggesting that these two species may contribute the most to the 3-dehydro-CA/CDCA blood fluxes. Note, however, that only six of the 116 pan-species metabolic reconstructions could produce 3-dehydro-CA/CDCA *in silico* (Table S9). Regressing the relative abundances of these two species against age found that *C. aerofaciens* weakly decreased with age (*p* = 0.12, Table S11), while such a decrease could not be found for *E. lenta* (*p* = 0.78). Our predictions of decreased 3-dehydro-CA/CDCA with age could not be validated against the serum metabolomic data, as these metabolites were not measured. Taken together, 3-dehydro-CA/CDCA blood production fluxes decreased with age, which was predicted to be mainly driven by *C. aerofaciens* abundances.

### Bile acid blood fluxes suggest APOE genotype to influence host-microbiome crosstalk

Next, we assessed the predicted fluxes against allelic variations in the *APOE* gene, which is the strongest genetic risk factor for AD^83^ and has been previously associated with compositional changes in the gut microbiota of humans^84,85^ and transgenic mice.^84,86,87^ *APOE* has three allelic variations, E2, E3, and E4.^88^ *APOE* E2 carriers are protected against AD,^89^ while E4 carriers have increased risk for AD.^90^ Given the six different allele combinations (E2/2, E3/3, E4/4, E2/3, E2/4, and E3/4) and the low abundance of the E2/2, E2/4, and E4/4 genotypes in our dataset (Table 1), we defined the following three *APOE* groups: E2, consisting of the lower risk *APOE* E2/E2 and *APOE* E2/E3 genotypes, E3, containing the normal risk *APOE* E3/E3 genotype, and E4, containing the higher risk *APOE* E3/E4 and E4/E4 genotypes. Note that we excluded 25 cases of the *APOE* E2/E4 genotype, as the allelic effect could not be defined. By performing Kruskal–Wallis tests^91^ on the predicted fluxes, we found significant associations with the *APOE* groups for the bile acid deoxycholate (*p* = 1.76.10^−2^, Table S14) and S-adenosyl-L-methionine (*p* = 3.25.10^−2^) before FDR correction for multiple testing.

**Figure 2.**
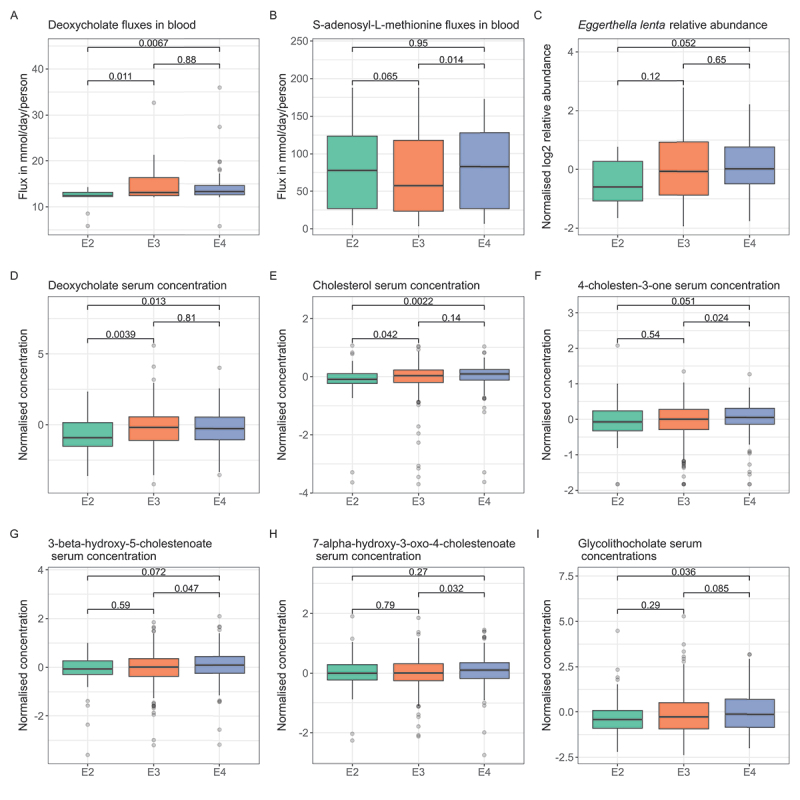
Overview of genetic associations with *APOE* groups.

Next, we performed pairwise multiple comparisons to find specific associations between these two metabolites and the three risk groups. The predicted deoxycholate blood flux values were found to be lower in the E2 group compared to both the E3 (*p* = 9.18⋅10^−3^) and E4 (*p* = 7.09.10^−3^) groups (Figure 2(A), Table S15). For S-adenosyl-L-methionine, the predicted blood fluxes were lower in the E3 group compared to the E4 group (*p* = 2.64⋅10^−2^, Figure 2(B)) and lower compared to the E2 group (*p* = 0.07) group. No difference in blood fluxes was observed between the E2 and E4 groups (*p* = 0.87).

A) Boxplots showing predicted blood fluxes in blood for deoxycholate in the E2, E3, and E4 groups. P-values were obtained through post hoc *Dunn’s* multiple comparison testing. The gray dots represent the deoxycholate blood flux values. An increase in deoxycholate flux is observed in the E4 group compared to E2. Panel B shows the predicted blood fluxes for S-adenosyl-L-methionine for the three groups. C) The normalized log2 relative abundances of *E. lenta* are shown for the *APOE* groups. Although missing significance, an increased relative abundance in the E4 group can be observed. D shows the normalized serum concentrations for deoxycholate. Here, increased concentrations of serum deoxycholate can be seen from E2 to E3 and from E2 to E4. E, F, G, H, and I all show serum concentrations of cholesterol and its downstream metabolites, including the secondary bile acid glycolithocholate. The compounds all show significantly or almost significant increases in serum concentrations from either the E2 to E3 groups or from the E3 to E4 groups.

We then tested whether these results could be explained by differences in the relative abundances of microbial species that could contribute to the predicted deoxycholate and S-adenosyl-L-methionine blood fluxes (Table S9, Table S10). The main microbial contributors to deoxycholate were *E. lenta* ($R^{2}$= 0.86) and *Bifidobacterium longum* ($R^{2}$= 0.18). For these species, no significant association was found with *APOE* risk groups (Table S16) using the Kruskal–Wallis test. A post hoc Dunn’s test, however, identified a weak increase in *E. lenta* abundance from the E2 to the E4 group (*p* = 0.1, Figure 2(C), Table S17). For S-adenosyl-L-methionine, none of the microbial species that could influence this compound correlated strongly with the predicted S-adenosyl-L-methionine blood fluxes. The relative abundances of *Butyricimonas virosa* and *B. vulgatus*, the species that best correlated with S-adenosyl-L-methionine blood fluxes, had $R^{2}$ values of 0.09 and 0.08, respectively (Table S10). When assessing the relative abundances of *B. virosa* and *B. vulgatus* against the *APOE* risk groups, no associations could be found (Table S16).

To determine if the predicted microbiome-driven flux associations could be captured in the serum metabolomics, a Kruskal–Wallis test and a post hoc Dunn’s test were performed on serum deoxycholate concentrations against the *APOE* risk groups. S-adenosyl-L-methionine was not present in the serum metabolomic data (Table S12). The serum deoxycholate concentrations were associated with the *APOE* risk groups (*p* = 1.31⋅10^−2^, Figure 2(D), Table S18). Lower deoxycholate serum concentrations were found in the E2 group compared to both the E3 (*p* = 4.08⋅10^−3^, Figure 2(D), Table S19) and E4 (*p* = 7.55⋅10^−3^) groups, validating the findings from the blood flux predictions. Note that no links between the predicted fluxes and weighted polygenic risk scores for AD could be found (Table S8, Methods). Taken together, deoxycholate levels were increased with the *APOE*-based risk factor for AD in both the predicted fluxes, i.e., gut microbiome-driven metabolic influences and in the serum metabolomic data. Enriched *E. lenta* abundances were identified to be a major influencer of predicted deoxycholate levels. As deoxycholate is known to be microbially derived,^49^ this result suggests a potential link between *APOE* genotype and an altered gut microbiome composition that includes enrichment of *E. lenta*.

### Serum metabolomics links APOE E4 with increased flux through cholesterol and bile acid metabolism

As deoxycholate is a downstream metabolite of primary bile acids and cholesterol,^49^ we hypothesized that measured serum concentrations of these upstream metabolites of deoxycholate might also be increased in higher risk *APOE* E4 individuals and decreased in lower risk *APOE* E2 individuals. This hypothesis was tested by associating cholesterol and its downstream products in the serum metabolomic data with the *APOE* risk groups. The measured serum metabolomic data contained 30 compounds involved in cholesterol and bile acid metabolism (Table S12). After associating the measured serum concentrations with the *APOE* genotype groups, significant associations were found for cholesterol (*p* = 6.08⋅10^−3^, Figure 2(E), Table S18) and its downstream derivative 4-cholesten-3-one (*p* = 3.04⋅10^−2^, Figure 2(F)). Furthermore, associations were found for the cholesterol products 3β-hydroxy-5-cholestenoate (*p* = 0.26, Figure 2(G)) and 7α-hydroxy-3-oxo-4-cholestenoate (*p* = 0.06, Figure 2(H)). A post hoc Dunn’s test further revealed that both cholesterol and 4-cholesten-3-one had increased measured serum concentrations from the E2 to the E3 group and from the E3 to the E4 group (Table S19). Both 3β-hydroxy-5-cholestenoate and 7α-hydroxy-3-oxo-4-cholestenoate were found to have higher measured serum concentrations in the E4 compared to the E3 group (Figure 2(G,H)). Lastly, we investigated if serum concentrations of secondary bile acids were associated with the *APOE* genotype and found glycolithocholate to be correlated with *APOE* (*p* = 0.09, Figure 2(I), Table S18).

To summarize, a targeted analysis of the measured serum metabolites in the cholesterol and bile acid pathway found significant concentration increases for cholesterol and 4-cholesten-3-one from the lower risk E2 to normal risk E3 groups and from the E3 to E4 groups. Weaker associations were found for the cholesterol derivatives 7-α-hydroxy-3-oxo-4-cholestenoate and 3β-hydroxy-5-cholestenoate and for the secondary bile acid glycolithocholate. Note that these metabolites were not in our predefined and manually curated metabolite list (Table S4) and thus, were not evaluated through *in silico* predictions. Our results suggest a potential metabolic link between *APOE*, cholesterol, and bile acid metabolism.

### *E. lenta* drives deoxycholate and lithocholate blood fluxes and is negatively associated with global cognition

Next, we investigated potential associations between the predicted metabolic fluxes and global cognition. A score for global cognition was constructed by performing a principal component analysis on a battery of general cognitive tests (Table 1, Methods) and taking the first principal component as previously described.^92^ The tests measured a variety of cognitive functions, including processing speed, attention, verbal learning, verbal memory, dexterity, and fine motor skills (Methods). Previously, global cognition scores have been found to be more sensitive compared to MMSE scores in identifying subtle cognitive changes in middle-aged cohorts.^92,93^ The constructed global cognition score captured 47% of variation within the cognitive test scores, which is similar to other cross-sectional studies using global cognition scores.^92,94–96^ Note that global cognition scores are dimensionless, meaning that lower or higher global cognition scores should be interpreted as relative cognitive differences within the cohort.

**Figure 3.**
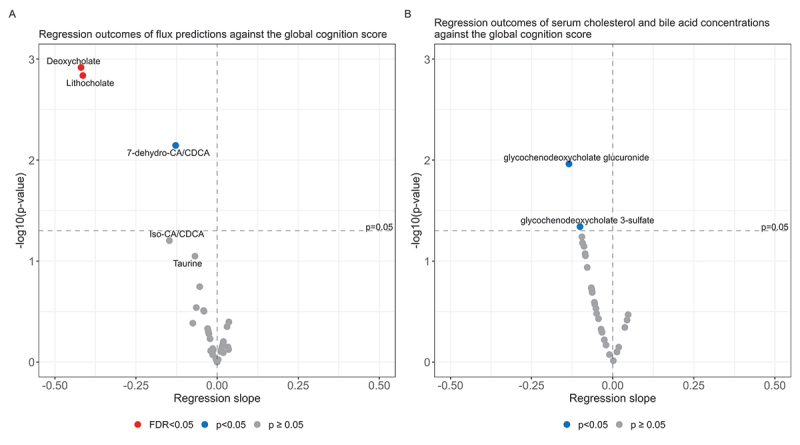
Overview of the predicted flux and serum metabolomic associations with the global cognition score.

After constructing the global cognition score, linear regressions were performed on the predicted blood metabolic fluxes against this score. Control variables were added for age, sex, BMI, and highest obtained education level. We found negative associations for deoxycholate (*p* = 1.21⋅10^−3^, FDR = 2.98⋅10^−2^), lithocholate (*p* = 1.46⋅10^−3^, FDR = 2.98⋅10^−2^), and for the metabolite group, 7-dehydroxycholate/7-dehydroxychenodeoxycholate. (7-dehydro-CA/CDCA, *p* = 7.17⋅10^−3^, FDR = 9.80⋅10^−2^, Figure 3(A), Table S8). These results suggest a lower global cognition score to be linked with higher predicted blood metabolic fluxes for these bile acids.

A) Volcano plot summarizing the regressing outcomes for all41 analysed flux distributions. The x and y axes, respectively, show the regression estimates and the −log10 transformed regression p-values. The estimates represent the coefficients of relative change in predicted flux with an increasing global cognition score, meaning that positive estimates coupled with significant p-values indicate increased microbiome flux capacities with the global cognition score. The red dots for deoxycholate and lithocholate show a negative regression estimate with global cognition scores coupled to an FDR-corrected p-value below 0.05. These results indicate increased flux capacities for these compounds increased with decreased global cognition scores. The blue coloured dots for the 7-dehydro-CA/CDCAs show a negative estimate coupled with a nominal p-value below 0.05, i.e., 7-dehydro-CA/CDCA blood fluxes nominally increased with a decreased global cognition score. All metabolites with regression p-values below 0.1 are labelled. The volcano plot in B) summarises the regression estimates of all metabolites involved in the cholesterol degradation pathway, primary bile acid metabolism, and secondary bile acid metabolism. Glycochenodeoxycholate glucuronide was the only serum metabolite that associated with the global cognition score, as indicated by the blue colored dot.

By tracing back the main microbial drivers of these fluxes, we found that *E. lenta* perfectly explained the predicted lithocholate blood fluxes ($R^{2}$ = 1, Table S10), in addition to its high explanatory value for deoxycholate blood fluxes ($R^{2}$ = 0.86). This perfect correlation with lithocholate means that *E. lenta* was the only species that produced lithocholate in the microbiome-WBMs and that host metabolism on potential microbially derived precursors did not influence the correlation. This result is likely caused by limitations in the sensitivity of 16S-RNA metagenomics and the reduced diversity due to the mapping procedure, as various species in the *Clostridiaceae* family, including *C. hiranonis*, *C. hylemonae*. and *C. scindens*, are also known to produce lithocholate.^51,67^ *Bacterioides ovatus*, a major driver of the predicted 7-dehydro-CA/CDCA blood fluxes, was found to be weakly associated with the global cognition score (*p* = 0.09, Table S11). This result can be interpreted as *B. ovatus* being a major driver of the negative associations with 7-dehydro-CA/CDCA blood fluxes. However, when performing linear regressions on the measured serum deoxycholate concentrations against the global cognition score, we could not find any associations (*p* = 0.82). This negative result could mean that 1. other species than *E. lenta*, not captured by our models, also produced deoxycholate *in vivo* and that 2. host bile acid synthesis differed between the individuals in the cohort. Inter-individual variations in host bile acids were not captured by our microbiome-WBMs due to the absence of personalised data, e.g., genomic variance in the associated genes. Additionally, 3. external factors that influence deoxycholate serum concentrations *in vivo*, such as diet and exercise could also explain this lack of agreement with our model predictions. Note that lithocholate and 7-dehydro-CA/CDCA were not measured in the serum metabolome (Table S12) and thus could not be compared with our predictions.

Interestingly, targeted regressions on bile acids and cholesterol metabolites in the serum metabolomics found weak negative associations across both primary and secondary bile acid metabolism (Figure 3(B), Table S13). Although significance was only achieved for the measured glycochenodeoxycholate glucuronide serum levels (*p* = 1.09⋅10^−2^), the regression coefficients for 24 out of 30 measured bile acids and cholesterol compounds were negative, which qualitatively aligned with our metabolic flux predictions (Figure 3(B)).

To summarize, the predicted blood metabolic flux predictions identified higher microbial production potentials for secondary bile acids in individuals with lower cognitive functioning. *E. lenta* was found to be the main driver of deoxycholate and lithocholate and was also enriched in individuals with lower global cognition scores. A targeted metabolomics analysis of cholesterol and bile acid compounds further identified small but consistently increased serum concentrations in individuals with lower global cognition scores. These results together suggest that lower global cognitive functioning in aging individuals is associated with metabolic and gut microbiome changes that resulted in altered secondary bile acid metabolism.

### Male exclusive flux associations were found with global cognition

Sex is another risk factor for AD and for declining cognitive health.^97^ Hence, we investigated whether sex-specific differences in predicted host-microbiome co-metabolism could be linked to cognitive health. First, sex differences in microbial abundances in the 16S rRNA sequencing data were analyzed at the phylum level. We found a significant association of sex only with the Verrucomicrobiota phylum (*p* = 0.03, FDR = 0.18, Table S20), which had an average read abundance of only 2.2% (SD = 5.38%). However, at the species level, several significant differences could be found (Table S21), with *Anaerostipes hadrus* (*p* = 4.09⋅10^−6^, FDR = 2.41. 10^−4^) having the largest differences in their relative abundances. When associating the predicted metabolic flux data with sex, we found 41 (59%) metabolites and metabolic groups had significantly different predicted flux values in the male cohort compared to the female cohort, i.e., FDR<0.05 after performing a Wilcoxon rank sum test^91^ (Table S22). Although the sex differences in predicted blood metabolic fluxes may also be a direct consequence of different parameterization and reaction content of the male and female WBMs,^65^ our sex-specific predicted metabolic flux differences could also result from the differences in microbial composition.

**Figure 4.**
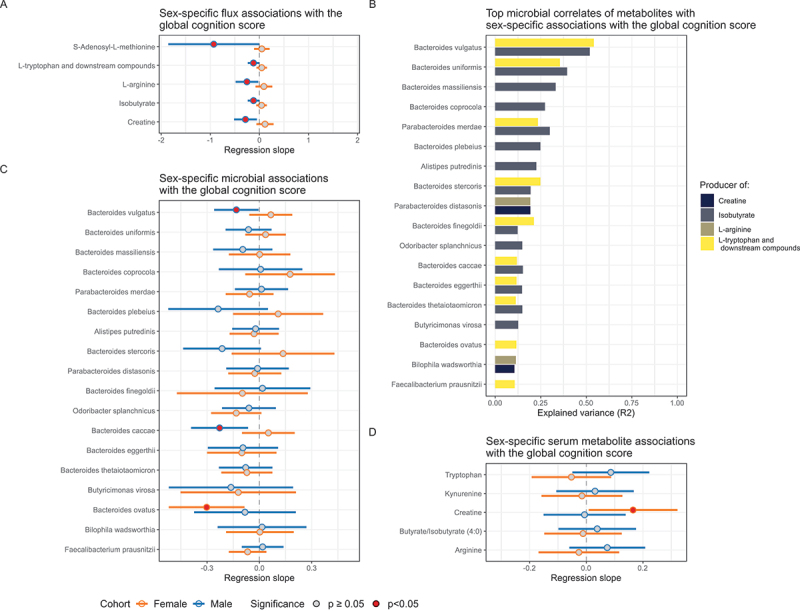
Integrated analysis of sex-specific predicted flux associations with global cognition scores.

To test whether sex-specific microbial influences may be linked with cognitive health, we performed a set of regression analyses based on previous work by Arnold and colleagues.^98^ We first performed linear regressions on the interaction term between the predicted metabolic flux and sex variables against global cognition. The interaction term indicated how much the regression slopes of the predicted blood fluxes against the global cognition scores differed between male and female microbiome-WBMs. Control variables were also added to account for age, BMI, and the highest obtained education level. Five metabolites were found to have significant (p<0.05) interaction terms (Table S23), being creatine, L-arginine, S-adenosyl-L-methionine, L-tryptophan and its degradates (flux group: L-tryptophan, L-kynurenine, serotonin, and indole-3-acetate), and isobutyrate. Note that none of these metabolites were significantly associated with the global cognition score in the entire set of 1,065 microbiome-WBMs. To assess whether sex-dependent associations existed for these five metabolites/metabolite groups, linear regressions against the global cognition score were performed separately on the predicted fluxes in the 457 male and 608 female microbiome-WBMs. These regressions yielded male-specific negative nominal associations with the global cognition score (Figure 4(A), Table S23), meaning that lower global cognition scores correlated with increased metabolic blood fluxes for these metabolites in male, but not in female models. No FDR adjusted significant associations were found.

A) Forest plot of regression outcomes for metabolic blood fluxes that were associated with global cognition in a sex-specific manner. Horizontal lines for the 95% confidence intervals are shown for the female cohort (orange) and the male cohort (dark blue). The regression estimates are indicated by the gray and red dots, with the gray colored dots indicating a regression p-value at or above 0.05, and the red dots indicating regression p-values below 0.05. Male-dependent negative associations with global cognition are shown for all compounds with sex-specific associations. B) Bar plot of all species that explained a minimum of 10% of variance in the blood fluxes of all compounds with sex-specific associations with global cognition and contained metabolic reactions for the production of these compounds. L-tryptophan and isobutyrate are both highly correlated with *B. vulgatus* and *B. uniformis*. L-arginine or creatine further correlated moderately with *P. distasonis* and *B. wadsworthia*. None of the species that could produce the S-adenosyl-L-methionine precursor, L-methionine, explained more than 10% of variance in S-adenosyl-L-methionine blood fluxes. C) Forest plot of sex-specific regression outcomes on relative species abundances against the global cognition score. Inclusion criteria for the species were identical to panel B. No sex-specific associations were found with relative species abundances, except for *B. vulgatus* and *B. caccae*, both of which are tryptophan and isobutyrate producers. D) Forest plot of sex-specific serum metabolomic associations with the global cognition score. Only metabolites that were predicted to be significantly associated with the global cognition score in males are shown. None of the analyzed serum metabolomic concentrations were linked with the global cognition score in neither males nor females.

For these five metabolites/metabolite groups, we identified species that best correlated with the predicted metabolic fluxes (Figure 4(B), Table S10) by performing the same set of linear regressions as with the predicted blood fluxes and the species abundances. These regressions found a male-dependent negative association (Table S24) for *B. vulgatus* (interaction term; *p* = 0.02) and *Bacterioides caccae* (interaction term; *p* = 0.01, Figure 4(C)), which both produced and correlated with L-tryptophan and isobutyrate (Figure 4(B)). Additionally, the L-tryptophan and isobutyrate producer *B. ovatus* was found to decrease in female samples (*p* = 6.78⋅10^−3^, Figure 4(C)). However, the regression slopes of *B. ovatus* relative abundances against the global cognition score did not significantly differ between male and female samples (*p* = 0.27).

We then tested whether the significant male-dependent associations of the predicted blood fluxes against the global cognition score were found in the measured serum metabolomic data. Regressions were performed for creatine, L-arginine, L-tryptophan, and kynurenine, as these compounds were also measured in the serum metabolomics (Table S12). No sex-specific influences were found for these compounds (Figure 4(D), Table S25), suggesting that the predicted sex-specific microbial links with the global cognition score did not significantly influence their serum concentrations *in vivo*, but again host metabolic differences and external factors masking any significant microbially caused differences could not be excluded.

To summarize, stratifying regressions on the predicted blood metabolic fluxes by sex resulted in significant influences of sex on the associations of the global cognition score with creatine, L-arginine, S-adenosyl-L-methionine, L-tryptophan and its degradation products, and isobutyrate. Predicted blood fluxes for these metabolites were further found to be significantly higher with lower global cognition scores in males, but not in females. These results could, however, not be replicated in the serum metabolomics, suggesting additional factors affecting the serum concentrations of these metabolites.

## Discussion

In this study, we systematically explored potential links between host-microbiome co-metabolism and risk factors of AD. Metabolic flux contributions of the gut microbiome were investigated 63 metabolites in blood, represented by 41 unique flux distributions in a cross-sectional cohort of 1,065 individualized microbiome-WBMs. The flux predictions were tested for correlations with age, *APOE* genotype, global cognition, and sex, and were further contextualized with correlation analyses on relative species abundances. Finally, metabolites with fluxes that associated with the tested AD risk factors were used to contextualize the flux results and inform targeted assessments of serum metabolomics against the AD risk factors. Our key results include i) an increased microbial capacity with age to produce L-arginine, ii) *APOE* associations with cholesterol and bile acid metabolism, and *E. lenta* abundances, iii) cognitive correlations with secondary bile acids, and iv) male-dependent correlations between global cognition and host-microbiome co-metabolism. Together, our findings support the crucial role for the gut microbes in cognitive health by linking predicted and measured metabolic phenotypes along the host-microbiome co-metabolic axis with risk factors of AD. Furthermore, our study highlights the importance of computational metabolic modeling to gain novel insight into microbial metabolites and associated host metabolism in cognitive health.

We predicted an increased microbial capacity with age to supply human metabolism with L-arginine (Figure 1), which would lead to age-related host-microbial changes in arginine metabolism. In the serum, a weak negative correlation with age was found, although the correlation was not significant after correction for multiple testing (Figure 1). Arginine levels have been found to increase in healthy individuals with age.^99,100^ Although the role of arginine and healthy aging in humans remains largely unclear, mice studies have repeatedly linked arginine metabolism to age-related cognitive decline.^101–104^ Increased arginine breakdown into the polyamines, spermine and putrescine, and the neurotransmitter glutamate, has been linked to decreased brain amyloid-β load in transgenic mice,^103^ suggesting a neuroprotective role of arginine and its downstream products in aging. Gut microbial productions of arginine have also been linked to healthy aging in a mouse model by He et al. (2024).^104^ He and colleagues demonstrated that the arginine producer *Akkermansia muciniphila* influenced the dysregulation of arginine metabolism in cognitively impaired mice and found that age-related cognitive impairments could be rescued by dietary provision of *Amuc_1100*, an *A. muciniphila* protein and arginine source. We could however not identify a clear correlation between the arginine-producing species and age or cognition in the Rotterdam cohort, likely due to the lack of cognitively impaired individuals. Nevertheless, the two species that best correlated with L-arginine fluxes, *P. distasonis* and *B. wadsworthia*, have been previously linked to normal aging.^105^ To summarise, our findings suggest that L-arginine metabolism in healthy aging is influenced by age-related global gut microbiome alterations.

Older age was also associated with predicted decreased fluxes of 3-dehydro-CA and 3-dehydro-CDCA (Figure 1). To our knowledge, this is the first study suggesting a link between age and microbial production of these compounds. Sixteen strains are known to produce these compounds,^51^ including six species captured in the microbiome-WBMs (Table S9). Our analysis found *C. aerofaciens* to be the main producer of 3-dehydro-CA and 3-dehydro-CDCA. Interestingly, this microbial species has been previously identified as a risk factor for AD,^106^ thus implicating *C. aerofaciens* in cognitive decline. Other 3-dehydro-CA/CDCA-producing strains associated with AD are *Gordonibacter pamelaeae*,^107^ which was also present in the microbiome-WBMs (Table S9) and *Collinsella stercoris*, which was not present in the microbiome-WBMs, but has been found to have increased relative abundance in AD patients.^29^ Taken together, although this result requires further experimental confirmation, it indicates gut microbial influences on healthy aging via bile acid converting microbes.

We also found associations between the predicted metabolic fluxes and *APOE* allele presence. Deoxycholate fluxes were lower in individuals with *APOE* E2 (Figure 2(A)), which was confirmed in the serum metabolomics (Figure 2(D)), Further metabolomic analysis found the microbe-derived bile acid glycolithocholate to be associated with *the APOE* genotype (Figure 2(I)). *E. lenta* was identified as the main deoxycholate producer in the microbiome-WBMs and was also associated with *the APOE* genotype (Figure 2(C)). A result that was also found in a Chinese population of community-dwelling older adults.^108^ More broadly, our findings on flux associations with *APOE* genotypes are in line with growing evidence of a potential genetic influence of *APOE* on gut microbiome compositions.^84–87,109^ However, our flux and metabolomic results also suggested that *APOE*-dependent gut microbiome alterations potentially result in altered productions of secondary bile acids. Such an interpretation should, however, be approached with caution, as confounding factors might explain the observed associations between secondary bile acids and *APOE* genotype. For example, prodromal AD features, such as mild cognitive impairment, are known to be associated with increased microbial bile acid conversion.^26^ At the same time, our results might also be explained by *APOE*-dependent altered primary bile acid synthesis,^109,110^ as bile acids have known antimicrobial effects and can influence the structure of microbiome communities.^45,46^ Nevertheless, future studies are needed to confirm a connection between *APOE* genotype and gut microbial enrichment of bile acid producing bacteria, such as *E. lenta*.

Our metabolomic measurements further found *APOE*-dependent differences in the metabolomic serum concentrations for cholesterol and its products, which are metabolic sources for bile acid synthesis.^49^ We found higher metabolomic cholesterol levels in *APOE* E4 carriers compared to *APOE* E2 carriers (Figure 2(E)). This *APOE* allele-dependent association was also found for the measured cholesterol degradation products: 4-cholesten-3-one (Figure 2(F)), 3beta-hydroxy-5-cholestenoate (Figure 2(G)), and 7-alpha-hydroxy-3-oxo-4-cholestenoate (Figure 2(H)). These findings are in line with known mechanistic effects of the ApoE protein isoforms on cholesterol metabolism, as reviewed elsewhere.^111,112^ Specifically, ApoE E2 is known to promote reduced blood cholesterol levels,^113^ while ApoE E4 promotes increased blood cholesterol levels.^114,115^ While our results on bile acid and cholesterol metabolism cannot establish a link between *APOE* genotype-dependent cholesterol breakdown and secondary bile acid production, the observation of *APOE*-dependent associations with both secondary bile acids and its metabolic precursors, cholesterol metabolites, suggests the possibility of a connection between *APOE*, cholesterol metabolism, and gut microbial bile acid producers. However, such a link remains to be experimentally validated.

Interestingly, associations with microbe-derived bile acids were also found for the global cognition score. Deoxycholate, lithocholate, and 7-dehydro-CA/CDCA fluxes were increased with lower global cognitive functioning (Figure 3). Secondary bile acids have been found to have cytotoxic^116^ and neuroprotective properties.^117^ Unconjugated bile acids can have cytotoxic effects,^118,119^ particularly deoxycholate and lithocholate.^116,120,121^ Previous metabolomic, transcriptomic, and COBRA modeling studies have found these compounds to be increased in both AD and mild cognitively impaired individuals.^26,39,43^ However, when we analyzed the serum concentration of these bile acids, we could not replicate our flux predictions with sufficient statistical confidence. Nevertheless, our modeling results on deoxycholate and lithocholate did inspire a further targeted metabolomic analysis of serum bile acids, which consistently found slightly higher bile acid serum concentrations in individuals with lower cognitive functioning (Figure 3), indirectly corroborating the metabolic flux predictions. Our results on increased microbial production of bile acids in lower cognitive health are in line with previous findings on microbial bile acid production in mild cognitively impaired and AD individuals.^26,43^ Our analysis of the microbiome samples further identified *E. lenta* and *B. ovatus* as the major drivers of deoxycholate, lithocholate, and 7-dehydro-CA/CDCA (Table S10). *E. lenta* enrichment is known to be associated with cognitive impairment,^108,122^ and also with AD.^123^ Our study, however, is the first to associate *E. lenta* to cognitive functioning in healthy individuals. Although enrichment of bile acid producing bacteria, such as *E. lenta*, in lower cognitive health should be confirmed in future studies, the replicated associations with both genetic and cognitive risk for AD provide some confidence in a potential link with AD pathogenesis.

Our analysis also found male-dependent correlations between predicted blood fluxes and global cognition for creatine, L-arginine, S-adenosyl-L-methionine, isobutyrate, and L-tryptophan (Figure 4). Although these results were not significant after multiple testing corrections and could not be replicated in the serum metabolomics, they might hint at potential sex-specific links between cognitive health and metabolic alterations along the gut-brain axis. Interestingly, male-dependent correlations with the global cognition score could not be replicated in the abundance data of species that produced creatine, L-arginine, S-adenosyl-L-methionine, isobutyrate, and L-tryptophan *in silico*. This lack of strong microbial correlates with the fluxes could be interpreted as the fluxes being emergent results of a multitude of small microbial variations across the gut microbiome. Sex-specific microbial effects on cognitive health have been previously described in various mouse studies.^124–126^ A recent study by Cuervo and colleagues^126^ on wild-type and AD transgenic mice has found lower abundances of microbial genes involved in arginine and proline metabolism in wild-type male mice compared to female mice. For transgenic AD mice, they found decreased abundances of microbial genes involved in tryptophan biosynthesis have been found in males compared to females. Our findings on tryptophan and arginine blood fluxes are in line with findings by Cuervo et al., suggesting the existence of male-specific microbial influences on cognitive health through tryptophan and arginine metabolism.^126^ Male-specific microbiome influences on AD pathology have also been found in other mouse studies.^124,125^ Both studies have found antibiotic-induced microbiome dysbiosis to result in a male-specific reduction of amyloid-β load in male mice with induced cerebral amyloid-β amyloidosis pathology. To summarize, although the sex-specific effects were small and were not found in the serum metabolomics, we believe that the topic of sex-specific metabolic trajectories along the gut-brain axis in preclinical dementia deserves further attention in future studies.

In this study, we also tested if the associations found by the flux predictions could be replicated in serum metabolomics. Most of the flux associations with the AD risk factors were not replicated in the serum metabolomics (see Figs. 1A–D and 4A–D). However, it is important to note that the fluxes and metabolomic concentrations measure inherently different features of whole-body metabolism. Predicted variations in the fluxes are influenced by compositional gut microbiome changes and their metabolic interactions with the host, while variations in serum metabolomic data can be influenced by a myriad of known and unknown factors, including host genetics, physiology, dietary intake, and environmental exposures. Additionally, both the metabolomic, metagenomic, and flux prediction data are influenced by different methodological biases, including sample collection, sample processing, and data generation. These differences limited the insights to be obtained from the flux–metabolomic comparisons. Nonetheless, the qualitative comparison between the flux and metabolomic statistical associations with AD risks provided a potential clue on the gut microbial influences on serum metabolite levels. For example, the associations of deoxycholate with *APOE* risk groups for the flux and metabolomic data provided an additional conformation of the modeling prediction that altered deoxycholate blood levels could be driven by compositional gut microbiome changes. Note that this modeling informed approach of testing metabolomic concentrations avoided the need for large-scale untargeted metabolomic investigations where small effects, like those found for serum cholesterol and bile acid compounds, might otherwise have been missed due to multiple testing penalties.

### Strengths and limitations

The strength of our study lies in its integration of metabolic knowledge bases, i.e., the microbial species and WBM reconstructions, with a diverse set of data modalities, including metagenomic, metabolomic, cognitive, and demographic data. This large diversity of data types and data sources was leveraged to obtain in-depth and highly contextualized predictions of gut microbial metabolic correlations with known risk factors of AD. Another strength was the large size of the cohort, which enabled the detection of weak metabolic signals that are expected in healthy and relatively homogeneous populations. Despite these strengths, the interpretation of the results was hampered by various limitations. The main limitation of the study was the lack of individuals with significant cognitive decline. This lack of cognitively impaired individuals reduced the clinical relevance of the results, as a correlation with a risk factor of AD does not necessarily imply a link with AD. Another major limitation was the low resolution of the gut microbiome data due to the use of 16S rRNA sequencing, rather than shotgun metagenomic sequencing. This shortcoming led to an overall low species and read coverage, lowering the accuracy of the host-microbiome modeling predictions. We highly recommend future investigations using higher resolution shotgun metagenomic sequencing to increase the mapping coverage and thus confidence in metabolic predictions. Another improvement could be made by using the recently introduced PANERA framework^127^ for generating hybrid genus-species metabolic models. Hybrid models may improve the prediction accuracy by incorporating more information from the gut microbiome data but also bear the risk of overestimating metabolic capabilities. Furthermore, the flux predictions were limited by inherent limitations of COBRA modeling, such as the steady state assumption, which consequently does not allow for capturing the dynamics of the human host–microbiome co-metabolism. Another limitation was that no causal links could be made between the metabolomics and the fluxes or species abundances, meaning that the similar associations between the fluxes and serum metabolomics could be explained by unknown confounding factors. Future studies could work around this limitation using the recently introduced *in silico in vivo* pattern analysis framework,^74^ which enables the investigation of causal links between gut microbiome abundances and metabolite concentrations.

## Conclusion

In conclusion, we identified gut microbiome influences on arginine and bile acid metabolism to correlate with risk factors of AD. No strong correlation was found between a single species and the AD risk factors, suggesting that these metabolic correlations are emergent properties of microbe–microbe and host–microbiome interactions. Furthermore, sex-specific correlations with global cognition were predicted for tryptophan and its degradation products, creatine, and S-adenosyl-L-methionine. Taken together, this work shows the gut microbiome to be connected with the main risk factors of AD. We hope our findings will inspire further investigations into gut microbial influences on metabolic shifts in preclinical AD.

## Methods

### Participant recruitment

This study used previously generated datasets of 1,065 participants in the RS-III-2 cohort within the Rotterdam study, a large-scale prospective study established in 1990 that periodically follows middle-aged and elderly individuals from the Ommoord area in Rotterdam.^80^ Recruitment for this cohort took place from February 2006 to December 2008. Participants were included if they met the inclusion criterion of being aged 45 or older at the time of recruitment and were not part of a previous cohort, and lived in the study district. All materials for this study have been produced between March 2012 and June 2014.

### Cognition scores

The global cognition metric was constructed as described by Hoogendam et al. (2014)^92^ and was based on five general cognitive tests. The following tests were included. The 15-word learning test,^128^ which measures verbal learning, immediate recall, and delayed recall. The Stroop color naming task^129^ is a reading and color naming inference test that measures processing speed. The letter-digit substitution test^130^ measured processing speed by writing numbers below corresponding letters. Furthermore, a word fluency test for categorising animals was included,^131^ which measured the speed of searching in long-term memory. Finally, the Purdue pegboard test, which measured fine motor skills and dexterity by letting the participant place pins in parallel rows with both hands simultaneously for 30 seconds.^132^ The cognitive scores were standardized and a single metric of global cognition was then constructed by taking the first principal component upon completing principal component analysis.^92^ Global cognition explained 47% of variance in the cognitive tests, which is similar to previous constructions of global cognition.^133,134^ Note that the global cognition score is sometimes referred to as the G-factor. Cognitive test scores and global cognition scores are shown in Table 1.

### Genotyping

Genotyping was performed from serum samples using polymerase chain reaction and a bi-allelic TaqMan assay for SNPs rs7412 and rs429358 as previously described by Licher et al. (2019).^135^ Imputation was carried out with the Haplotype Reference Consortium reference panel (version 1.0) using Minimac 3.

### Polygenic risk scores

The polygenic risk scores were constructed as described by Licher et al. (2019) from a genotyping study of 28 single nucleotide polymorphisms (SNPs, Table S26 for the list of included SNPs) as follows:

$$\mathrm{PRS}=\overset{j}{\underset{i=1}{\sum }}D_{i}\times E_{i},$$

with D being the allelic dosage (number of compounds measured of a given SNP) for SNP *i*, and E being the fitted regression coefficient of how an SNP associated with AD for SNP *i*. The fitted regression coefficients for each SNP have previously been determined and validated by Licher et al (2019).^135^ *J* represents the number of included genes.

### Metabolomics

Untargeted serum metabolomics was profiled on blood samples from 1,082 participants in the RS-2-III cohort using the Metabolon HD4 platformTM. The profiled samples contained 1,387 metabolites, including various amino acids, carbohydrates, cofactors, energy-related compounds, nucleotides, peptides, and xenobiotics. The provided Metabolon data wwere batch-normalized by dividing the sample peak intensity by the median value for each batch. After obtaining the data, 14 samples were excluded due to these samples missing values of over five times the standard deviation of the overall mean number of missing values. Next, metabolites were removed with missing values in over 70% of the samples, resulting in 120 metabolites being excluded. In addition, metabolites with high batch heterogeneity were removed by excluding compounds with a between aliquot coefficient of variance (CV, with CV=standarddeviation/mean) above 30%. After metabolite removal, 1,111 metabolites, of which 808 were named metabolites (Table S12), were available for analysis. All values were log2-transformed, and missing values were imputed using K-nearest neighbor imputation.^136^

### Gut microbiome data

The gut microbiome dataset has been previously described in Radjabzadeh et al.^82^ In short, the data for this study have been generated from fecal samples collected between March 2012 and June 2014 during the second checkup of the RS-III-2 cohort. Fecal samples have been collected at home, after which they have been sent by mail for analysis. Samples were not analyzed if the participant took antibiotics in the month before sample collection and if the mailing process to the Rotterdam study center took more than three days. Microbial abundances have been obtained by isolating the microbial DNA in each fecal sample and performing 16S gene sequencing of the V3 and V4 hypervariable regions using an Illumnia MiSeq sequencer. 16S read processing and taxonomic classification has been previously done by Radjabzadeh and colleagues,^82^ using an *in-house* processing pipeline (microRapTor) based on QIIME^137^ (version 1.9.0) and UPARSE^138^ (version 8.1).

### Mapping and processing of microbe abundances

Mapping of the microbiome data was performed using the MARS pipeline^139^ against the AGORA2 resource,^68^ which contains 7,302 high-quality refined genome-scale metabolic reconstructions of microbial strains known to inhabit the human gut. AGORA2 contains strain-specific metabolic reconstructions. Hence, they could not be directly used for mapping, as the microbiome data had a maximal resolution at the species level. To obtain the species included in AGORA2, the strain reconstructions were combined into pan-species reconstructions that include the union of all unique reactions, metabolites, and genes of the set of strains within a given species (*createPanModels.m*) from the Microbiome Modelling Toolbox^70^ within the CobraToolbox.^58^ The species level was chosen as it was the most precise taxonomic level in the microbiome dataset. Including higher taxonomies would have resulted in the inclusion of species with more diverse metabolic phenotypes that were not found in the microbiomes. After obtaining the pan-species reconstructions, the microbiome reads were filtered on species that were present in AGORA2. Each reaction in the pan-species is coupled through a coupling constraint (see below) to the corresponding biomass reaction, which enlists all known metabolites known to be required for producing a new cell (e.g., amino acids and nucleotides). Next, the microbiome data were further processed by calculating the relative read abundances for each individual.^139^ Therefore, we scaled the read counts such that the sum of read abundances equaled one. These relative abundances were processed by setting all reads with a relative abundance lower than 1e-6 to zero and subsequently normalizing the reads again.

### Reconstruction of microbiome community models

Microbiome community models were generated using the Microbiome Modelling Toolbox 2.0^70^ by running the *mgPipe.m* function. For each sample, pan-species reconstructions were connected to a shared microbiota lumen and reactions were added that enabled metabolites to be shared between pan-species reconstructions through the lumen. The relative abundances of the mapped microbial species per sample were integrated into the microbiome community models through a so-called community biomass reaction (VMH reaction ID: communityBiomass), which lists each microbe’s biomass reaction with the microbe’s relative abundance as the stoichiometric coefficient.^61^ This communityBiomass reaction was added to ensure proportional growth of each pan species subnetwork based on the measured relative abundances within the community model. Consequently, the contribution of each microbe to the host blood metabolites was proportional to its relative abundance and its metabolic activity within the host-microbiome WBMs.

### Sex-specific, organ-resolved whole-body models of human metabolism

For the human part of the microbiome-WBMs, we used generic male and female germ-free WBMs from^78^ (see Supplementary materials). These WBMs were extended by 1,933 reactions, 1,440 metabolites, and 1,735 constraints for the male WBM and 2,079 reactions, 1,490 metabolites, and 1,797 coupling constraints to the female WBM, respectively. The WBMs included 85,403 reactions 60,341 metabolites, and 109,148 coupling constraints across 30 organs for the female, and 82,913 reactions 57,897 metabolites, and 105,355 coupling constraints across 28 organs for the male WBM. No other changes were made to the parameterization of these generic WBMs.

### Creation of microbiome-WBMs

Microbiome personalized WBMs were generated as described by Thiele et al. (2020) using the *combineHarveyMicrobiota.m* function in the CobraToolbox. Briefly, this function combines the microbiome community models with the WBM hosts by connecting the microbiome community models to the WBM large intestinal lumen compartment, enabling metabolic crosstalk between the gut microbiota and the host. The male and female hosts and microbiome models were joined in a manner corresponding to the sex of the sample provider. A coupling (flux capacity^65,140^ constraint of 400 was used for the microbial part, which coupled each microbe’s reaction to its biomass reaction. After adding the microbiome models to the WBMs, the male microbiome-WBMs contained on average 131,910 (SD = 12,828) reactions, 100,662 (SD = 11,213) metabolites, and 175,956 (SD = 19,293) constraints. The female models’ microbiome-WBMs included 132,872 (SD = 11,999) reactions, 101,764 (SD = 10,453) metabolites, and 177,436 (SD = 18,002) constraints (Table 3). Each microbiome-WBM was constrained by setting the upper and lower flux bounds of the *Excretion_EX_microbiota_LI_biomass[fe]* reaction to one mmol/day/person. This reaction excretes the *microbiota_LI_biomass* compound, which is produced by the *communityBiomass* reaction. This constraint ensures that all microbiome-WBMs produce the same microbial community flux value, albeit the microbial community composition may be different. Similarly, the upper and lower flux bounds of the *Whole_body_objective_rxn* was set to one, as previously described by Thiele and colleagues in 2020.^65^ The *Whole_body_objective_rxn* contains a linear combination of the organ biomass productions scaled by their proportional weights. Constraining the *Whole_body_objective_rxn* ensured equal proportional organ biomass productions between samples corresponding to their weight maintenance.

### Diet formulation

The dietary uptake reactions of the microbiome-WBMs were constrained to an average European diet, taken from the Virtual Metabolic Human database (https://www.vmh.life).^62^ The average European diet was chosen, as no dietary information was available. Briefly, the diet formulation represented the molecular makeup of an average one-day meal plan for a 70 kg adult^62^ and was given as a metabolic flux rate in millimoles per person per day. The diet contained 97 metabolites; however, this formulation is likely to be incomplete as only metabolites that were measured as ingredients were included.^62^ The formulation for the average European diet was

### Curation of the metabolite list

A list of metabolites for the prediction of blood metabolic fluxes was generated by manually selecting compounds within the 1,032 metabolites that were present in the blood compartments of both the male and female WBM reconstruction. Metabolites with known involvement in cognitive health, AD, and the gut–microbiome, including 15 energy-related metabolites,^141^ 10 secondary bile acids,^43^ 10 neurotransmitters,^142^ 7 short-chain fatty acids,^143,144^ 7 compounds in tryptophan metabolism,^145,146^ 6 compounds in methionine and cysteine metabolism,^38,147^ 3 branched-chain amino acids,^36,37^ 3 polyamines,^38^ and 2 lipid compounds, were selected (Table S4).

### Simulations

Metabolic fluxes in the microbiome-WBMs were predicted using the CobraToolbox^58^ and the PSCM modeling toolbox.^65^ In the COBRA approach, the metabolic network represented a stoichiometric matrix *S*, where each row represents a metabolite *i* and each column a reaction *j*. Stoichiometric coefficients are given to the entries in *S* to indicate if a metabolite takes part in a reaction (non-zero entry) or not (zero entry). By definition, reaction substrates have a negative coefficient, while reaction products have a positive coefficient. Changes in metabolite concentration $x_{i}$ over time *t* are defined by *S* times a reaction flux vector *v* :^148^

$$\frac{\mathrm{dx}}{\mathrm{dt}}=S\cdot v$$

In COBRA modeling, a steady state is assumed, i.e., no change in metabolite concentration over time occurs:

$$\frac{\mathrm{dx}}{\mathrm{dt}}=S\cdot v\;\;\equiv \;\;0$$

Additional constraints are placed upon *v*, limiting the values that $v_{i}$ can take: a lower flux bound, *lb*, and an upper flux bound, *ub*. These flux bounds are based on thermodynamic and experimental data (e.g., from the literature). Reactions for which this information was not available were left unconstrained by setting *lb* and *ub* to arbitrary values of −1,000,000 and 1,000,000 mmol/person/day (see also Thiele et al.^65^ for details). Additionally, an objective function for the FBA problem is defined, indicated by non-zero values in *c*. The following linear programming problem was solved:

$$\underset{v}{\max}\;\;c^{T}\cdot v$$

s.t, S⋅v=0,

 lb≤v≤ub

The resulting flux distribution *v* contained a flux value for each reaction *j* in the microbiome-WBMs, or the germ-free sex-specific WBMs. We applied FBA to calculate the maximal possible metabolite flux rates in the blood compartment, as described elsewhere.^65^ Briefly, artificial demand reactions^58^ were added to the blood compartment for each of the selected metabolites using the *addDemandReaction.m* function from the CobraToolbox.^58^ Demand reactions are unbalanced left-sided reactions (e.g., DM_dchac[bc]: 1 dchac[bc] →Ø for deoxycholate in blood), effectively removing the corresponding metabolite from the system. The added blood demand reactions were sequentially set as an objective function and maximized using FBA. This approach has been successfully used to predict blood biomarkers associated with single-gene defects.^65,149^ Practically, FBA was performed using the *optimizeWBModel.m* function, which calls the function *FBA = optimizeWBModel(model)*. To ensure maximization of the objective function *model.osenseStr = “max”* was set. All default parameters for the LP solver were used.

### Flux processing

Before analyzing the fluxes, various transformations were performed to aid the interpretability of the statistical analyses. First, the fluxes were rounded to six decimals. Then, the flux values were scaled to find the net microbial influence on the fluxes in blood. The scaled flux values were calculated by subtracting fluxes obtained from germ-free WBMs from the predicted host-microbiome fluxes. As the germ-free WBMs were also parameterized with the average European diet, this operation removed the dietary contribution to the metabolite flux values. Variations in the scaled fluxes thus represented variations in host-microbiome co-metabolism, as the host effects were not removed from the scaled flux values. Further alterations to the predicted flux distributions were made by removing results that indicated no microbial contribution to the flux prediction, i.e, microbial flux contributions of zero. Moreover, fluxes that were limited by a host or dietary reaction flux-bound were identified by finding flux values in the cohort where the maximal obtained flux for a given metabolite occurred two or more times. These fluxes were removed, as these results would have caused a methodological bias in the statistical analyses of the flux distributions. Note that for the subsequent analysis, only the objective values, i.e., maximal possible flux value through each of the artificial demand reaction, were used.

### Refinement of metabolite selection

After processing the predicted blood fluxes, metabolites were removed when no non-zero flux values could be predicted in over 90% of samples or if identical flux values, within a tolerance of 1e-6 mmol/day/person, were obtained for 90% of the samples or more (Table S4). Next, metabolites were removed if their predicted maximal possible blood flux values were linearly dependent on the maximal possible blood flux value of an upstream metabolite, i.e., when two predicted metabolic flux values were stoichiometrically linked. To identify those, pairwise simple linear regressions were performed for each metabolite pair. Two metabolites were deemed linearly dependent and considered a group if >0.999. If the predicted maximal possible flux values of multiple metabolites could be linearly mapped to each other, but none of the metabolites were a precursor to all others in the group, only the flux values from the metabolite with the smallest maximal possible flux value were selected for further analysis. To illustrate this process, 3-dehydrocholate and 3-dehydrochenodeoxycholate had nearly identical fluxes (>0.999), but with a slightly smaller mean flux for 3-dehydrocholate. The flux values from 3-dehydrochenodeoxycholate were removed and 3-dehydrocholate was renamed to 3-dehydro-CA/CDCA.

### Statistical analyses

To assess potential associations between the predicted blood flux values and age, linear regressions were performed for age in years as the predictor and predicted maximal possible flux as the response variable. Additionally, a control variable for sex was added. Age and flux were both standardized before performing the regressions. Links between the blood fluxes and the global cognition score were investigated by performing linear regressions on the standardized blood fluxes as the predictor and the global cognition score as the response variable. Control variables were further added for age, sex, BMI, and education. Links between the blood fluxes and *APOE* genotypes were investigated by performing a non-parametric Kruskal–Wallis test and a post-hoc Dunn’s test for pairwise multiple comparisons. Sex-specific associations between the flux values and the global cognition score were investigated by performing linear regressions on the interaction term for flux and sex against the global cognition score as the response variable. Control variables for age, BMI, and education were added. For metabolites with a significant p-value, i.e., *p* < 0.05, linear regressions were performed on the global cognition score-stratified female and male individuals with control variables for BMI and education. All statistical analyses on the plasma metabolomic data and the relative species abundances were performed identically to the analyses on the flux data. The relative species abundances and serum metabolomics were log2-transformed and standardized via z-transformation before performing statistical analyses.

### Software

All modeling work, including the construction and analysis of the microbiome-WBMs, was done in MATLAB 2020b (Mathworks^TM^). In addition, the parallel computing toolbox, statistics and machine learning toolbox, and the bioinformatics toolbox were utilized to generate and analyze microbiome-WBMs. FBA was performed using the IBM ILOG CPLEX 12.10 linear solver (IBM Inc.). Data processing and all statistical analyses were performed in the R programming environment (v.4.4.0) using the tidyverse packages.^150^ The lme4 package^151^ was used to calculate the linear regressions and the 95% confidence intervals. CobraToolbox v3^58^ with its Microbiome Modeling^70^ and PSCM^65^ toolboxes were used.

## Supplementary Material

Supplementary Tables Hensen et al for resubmission to Gut Microbes Reports.xlsx

## Data Availability

All data can be accessed upon request by A. Ikram.

## Code availability

All scripts can be accessed at https://github.com/ThieleLab/CodeBase.
